# Human papillomavirus genotypes in human immunodeficiency virus-positive patients with anal pathology in Madrid, Spain

**DOI:** 10.1186/1746-1596-8-204

**Published:** 2013-12-10

**Authors:** Benjamín García-Espinosa, Ernesto Moro-Rodríguez, Emilio Álvarez-Fernández

**Affiliations:** 1 Department of Histology and Anatomical Pathology, Rey Juan Carlos University, School of Medicine, Madrid, Spain; 2 Department of Anatomical Pathology and Laboratories, Hospital General Universitario “Gregorio Marañón”, Madrid, Spain; 3 Universidad Rey Juan Carlos, Av de Atenas s/n, E28922, Alcorcón, Madrid, Spain

**Keywords:** Anal squamous intraepithelial lesions, Human papillomavirus, Polymerase chain reaction, Genotyping, HIV, Anus, Spain

## Abstract

**Background:**

We studied anal specimens to determine the distribution of human papillomavirus (HPV) genotypes and co-infection occurrence. This information will contribute to the knowledge of HPV genotype distributions and provide an estimate of the prevalence of different oncogenic HPV genotypes found in patients in Madrid (Spain).

**Methods:**

We studied a total of 82 anal biopsies from the Hospital General Universitario Gregorio Marañón of Madrid. These included 4 specimens with benign lesions, 52 specimens with low-grade anal squamous intraepithelial lesion, 24 specimens with high-grade anal squamous intraepithelial lesions and 2 specimens with invasive anal carcinoma. HPV genotyping was performed with PCR amplification and reverse dot blot hybridization.

**Results:**

We detected 33 different HPV genotypes, including 16 HPVs associated with a high risk of carcinogenesis, 3 HPVs associated with a highly likely risk of carcinogenesis and 14 HPVs associated with a low-risk of carcinogenesis. In two specimens, an uncharacterized HPV genotype was detected. The most frequent HPV genotypes found were HPV-16 (10.3%; 95% CI: 6.6%-15.1%), HPV-52 (8.5%; 95% CI: 5.2%-13%) and HPV-43/44 (7.6%; 95% CI: 4.5%-11.9%). HPV-18 was only detected in 0.9% (95% CI: 0.1%-3.2%) of the total viruses detected in all lesions. HPV co-infections were found in 83.9% of all types of lesions. The majority of cases (90.2%) were concomitantly infected with the human immunodeficiency virus (HIV).

**Conclusion:**

The prevalence of high-risk carcinogenic genotypes in anal pathological samples was remarkable. Therefore, further studies that include a greater number of samples, particularly invasive carcinoma cases are needed to evaluate the potential influence of these HPV genotypes in the appearance of anal carcinomas. Also, the influence of other accompanying infections should be evaluated clarify the appearance of this type of carcinoma.

**Virtual slides:**

The virtual slide(s) for this article can be found here: http://www.diagnosticpathology.diagnomx.eu/vs/2075238024106058.

## Introduction

Anal squamous cell carcinoma is an uncommon tumour. However, it is the most common type of cancer malignancy that arises from the anal margin. Less frequent anal malignancies include neoplasms from the anal canal, which have been poorly differentiated and are typically non-keratinizing, and malignancies that arise around the dentate line which have been defined as transitional carcinomas [1,2].

In Spain, anal carcinoma is estimated to represent 1.8% of all tumours of the digestive tract. However, in the last 25 years, its incidence has increased in Spain, as in other Western countries. This increased frequency has been attributed to different factors, including increases in human papillomavirus (HPV) infections and other sexually-transmitted infections, the pandemic of human immunodeficiency virus (HIV) and the increasingly common practice of anal intercourse, in both homosexual and heterosexual settings [3-6].

HPV is the most common sexually-transmitted infection world-wide. Different lesions range from simple warts or *condyloma acuminatum* to severe changes like epithelial dysplasia and invasive carcinoma. The presentation varies with the different specific HPV genotypes that can infect the anogenital or oral mucous membrane. Of 100 recognized HPV subtypes, over 80 have a completely sequenced genome to date; 35 are specific for the cervical and anal epithelium. Each subtype carries a variable capacity for causing dysplastic changes. A recent systematic review showed that 72% of anal invasive carcinomas were associated with the oncogenic subtypes, HPV-16 and HPV-18. Currently, the relationships between the different HPV subtypes and their corresponding effects on the anogenital epithelium are well known [7,8].

Patients in immunosuppressed conditions are at increased risk for developing multiple HPV-related benign and malignant anogenital tumours. Patients that are HIV-positive fail to clear HPV-related diseases because they have insufficient cell-mediated immunity to control latent HPV infections. As HIV/AIDS is increasingly managed as a chronic disease, greater attention should be focused on cancer screening and prevention, particularly for non-AIDS-defining cancers in the anus and liver, and for Hodgkin lymphoma, which appears to have increased incidence [9]. A recent meta-analysis estimated that the anal cancer incidence was much higher in men that were HIV-positive (46 per 100,000 per year) than in those that were HIV-negative (5 per 100,000 per year) [10]. At the same time, patients that receive organ transplants and follow chronic immunosuppressive treatments are at risk for anal cancer [11].

Premalignant changes have been classified as low-grade and high-grade anal squamous intraepithelial lesions (LASIL and HASIL, respectively), based on criteria for evaluating similar lesions in the cervix; however, the classifications, follow the lower anogenital squamous terminology (LAST). This two-tiered scale defines low-grade lesions as those generally related to self-limiting HPV infections and the high-grade lesions as those with potential for progression to invasive carcinoma (IC) [12,13]. ASILs are most frequently found in the anal canal and perianal skin, and multicentricity is common [14].

To date, no study has investigated the relationship between HPV and ASILs in Spain; furthermore the prevalence of HPV is largely unknown. In other European countries, few studies have been published that estimated the HPV genotype distribution in anal lesions, in populations that were either HIV-negative and HIV-positive; thus, further studies in this field are needed [15-20].

The present study aimed to determine the HPV genotype distribution and co-infection rate in anal specimens from individuals in Madrid (Spain). The findings presented in this paper will contribute to the knowledge of HPV genotype distribution and provide an estimate of the prevalence of oncogenic HPV types in anal lesions among Spanish patients.

## Materials and methods

### Specimen collection and diagnosis

This study had a cross-sectional and retrospective design. We obtained samples from 226 individuals with anal lesions diagnosed in the Hospital General Universitario “Gregorio Marañón” of Madrid, between February 2012 and March 2013. The hospital provided healthcare to a population of about 750,000 individuals (11.5% of the population of the regional community of Madrid).

All patients were enrolled in a screening program introduced in the hospital to detect early sings of anal carcinoma. These patients were routinely subjected to anal examinations, followed by cytology. Suspected cases underwent a proctoscopy and were examined under anaesthesia. Suspicious lesions, identified by acetic acid staining, were mapped and biopsied for histological confirmation, according to the recommendations of the European Society for Medical Oncology Clinical Practical Guidelines [21].

The present study includes samples of anal lesions (specimens of fixed tissue sections from biopsies) collected from different departments of the Hospital General Universitario “Gregorio Marañón” of Madrid and submitted to the anatomical pathology laboratory for study.

The study included 82 samples from patients with an anatomic-pathological diagnosis of anal HPV-related lesions. Of these 82 patients, 74 with HIV infections were selected to investigate the presence of HPV in corresponding anal lesions. In 6 of these cases, the presence of HPV was negative, and in 6 other cases, the anal specimens were inadequate for HPV testing; in 62 HIV-positive cases, HPV was detected in anal lesions (HIV&HPV). The latter 62 cases were studied further. The average age of these patients was 45.7 years, and the majority (n = 58; 93.5%) was male.

The HIV&HPV samples included 1 benign lesion (*condyloma acuminatum*), 40 LASILs (including flat condyloma, mild dysplasia and ASIL 1), 20 HASILs (including moderate dysplasia, ASIL 2, severe dysplasia, ASIL 3, and carcinoma in situ) and 1 IC.

Informed consent was not required for this study because the results were based on routine HPV genotyping, and the analyses were performed as an adjunct to the cytological and histological study, in an anatomic pathology laboratory. The detection and genotyping was performed in a clinical setting, and patient confidentiality was protected by deleting any data that could identify the patients; thus it was not possible to determine the ethnicity of each patient. The study was supervised by the Ethics committee of the hospital (Comité ético de investigación clínica).

### Detection and genotyping of HPV

DNA for each case was extracted from an anal, liquid-based, cytology sample. The isolated DNA was used as a template for PCR amplification to detect the presence of HPV DNA. We used primers specific for the GP5-6 L1 consensus region. PCR amplification was targeted to a fragment of 450 bp in the L1 consensus region. We performed reverse dot blot hybridization with probes specific for each genotype (based on “flow-through” technology). The amplification products were separated with agarose gel electrophoresis and stained with ethidium bromide, which allowed detection of the products with a UV transilluminator. A sample was considered positive when a band of 150 bp was detected (GenoFlow HPV Array Test Kit © Diagcor Bioscience Incorporation Limited, Hong Kong).

This diagnostic kit was intended for simultaneous screening and genotyping of 33 HPV types. These HPV types included 15 associated with a high-risk of carcinogenesis (HR; HPV-16, HPV-18, HPV-31, HPV-33, HPV-35, HPV-39, HPV-45, HPV-51, HPV-52, HPV-56, HPV-58, HPV-59, HPV-68, HPV-73 and HPV-82), 3 associated with a highly-probable-risk of carcinogenesis (PHR; HPV-26, HPV-53, HPV-66) and 15 associated with a low-risk of carcinogenesis (LR; HPV-6, HPV-11, HPV-40, HPV-42, HPV-43, HPV-44, HPV-54, HPV-55, HPV-57, HPV-61, HPV-70, HPV-71, HPV-72, HPV-81 and HPV-84).

This kit was not able to detect some genotypes separately; thus, some were detected as couples (HPV-40/61, HPV-43/44, HPV-54/55, HPV-57/71, HPV-66/68 and HPV-84/26).

Two methods were used to estimate the frequency of HPV positivity. First, the percentage of lesions infected by one or several genotypes; and second, the percentage of a given HPV in the total number of viruses detected in all lesions and each group of lesions.

### Statistical analysis

Statistical analysis was performed with Stata version 11.1/SE (StataCorp. LP, TX, USA). Relative frequencies of HPV genotypes were estimated as percentages with 95% confidence intervals (95% CI) assessed with Clopper-Pearson method and based on the exact binomial distribution of the tail areas.

## Results

### Distribution of viral genotypes

At least 83.8% of the entire group of HIV-positive specimens studied was also HPV-positive (HIV&HPV; 62/74). Concomitant infections were found in a large proportion of HIV&HPV samples. The most frequent concomitant infections were from *Treponema pallidum* (40.3%) and Hepatitis B and/or C virus (30.6%).

As shown in Table 1, among the total group of specimens, the most frequent lesion was LASIL (64.5%), followed by HASIL (32.3%). Only one IC was diagnosed (1.6%). HPV co-infection were found in 52 of 62 cases (83.9%).

**Table 1:** Frequency of anal lesions founded in HIV&HPV + cases

	Frequency*		
**Pathological diagnosis**	**Total HPV + cases**	**Single HPV infection cases**	**Multiple HPV infection cases**
**BL**	1 (1.6)	1 (1.6)	0 (0)
**LASIL**	40 (64.5)	8 (12.9)	32 (51.6)
**HASIL**	20 (32.3)	1 (1.6)	19 (30.6)
**IC**	1 (1.6)	0 (0)	1 (1.6)
**Total specimens**	62 (100)	10 (16.1)	52 (83.9)

* Number of cases and percentages referred to the total of specimens (62).

Based on all the data, we detected 33 different HPV genotypes, including 16 HR-HPVs, 3 PHR-HPVs and 14 LR-HPVs. As shown in Table 2, among all the viruses, HPV-16 was detected most frequently (10.3%; 95% CI: 6.6%-15.1%). This was followed by, in order of decreasing frequency, HPV-52 (8.5%; 95% CI: 5.2%-13%), HPV-43/44 (7.6%; 95% CI: 4.5%-11.9%), HPV-6 (6.7%; 95% CI: 3.8%-10.8%) and HPV-51 (6.3%; 95% CI: 3.5%-10.3%). HPV-18 was only detected in 2 lesions (0.9%; 95% CI: 0.1%-3.2%) among HIV&HPV positive samples.

**Table 2:** Distribution of HPV genotypes found in the study according to the pathological diagnosis

**Genotype found**	**Total lesions**					**LASIL**					**HASIL**				
	**N**	% *	**CI95%**	% **	**CI95%**	**N**	% *	**CI95%**	% **	**CI95%**	**N**	% *	**CI95%**	% **	**CI95%**
**HR-HPVs**															
**16**	23	10.3	6.6-15.1	37.1	25.2-50.3	12	8.5	4.5-14.4	30.0	16.6-46.5	10	12.7	6.2-22.0	50.0	27.2-72.8
**18**	2	0.9	0.1-3.2	3.2	0.4-11.2	0	0.0	0.0-2.6	0.0	0.0-8.8	2	2.5	0.3-8.8	10.0	1.2-31.7
**31**	13	5.8	3.1-9.8	21.0	11.7-33.2	7	5.0	2.0-10.0	17.5	7.3-32.8	6	7.6	2.8-15.8	30.0	11.9-54.3
**33**	6	2.7	1.0-5.8	9.7	3.6-19.9	2	1.4	0.2-5.0	5.0	0.6-16.9	4	5.1	1.4-12.5	20.0	5.7-43.7
**35**	5	2.2	0.7-5.2	8.1	2.7-17.8	3	2.1	0.4-6.1	7.5	1.6-20.4	2	2.5	0.3-8.8	10.0	1.2-31.7
**39**	5	2.2	0.7-5.2	8.1	2.7-17.8	4	2.8	0.8-7.1	10.0	2.8-23.7	1	1.3	0.0-6.9	5.0	0.1-24.9
**45**	8	3.6	1.6-6.9	12.9	5.7-23.9	6	4.3	1.6-9.0	15.0	5.7-29.8	2	2.5	0.3-8.8	10.0	1.2-31.7
**51**	14	6.3	3.5-10.3	22.6	12.9-35.0	10	7.1	3.5-12.7	25.0	12.7-41.2	4	5.1	1.4-12.5	20.0	5.7-43.7
**52**	19	8.5	5.2-13.0	30.6	19.6-43.7	13	9.2	5.0-15.3	32.5	18.6-49.1	6	7.6	2.8-15.8	30.0	11.9-54.3
**56**	5	2.2	0.7-5.1	8.1	2.7-17.8	4	2.8	0.8-7.1	10.0	2.8-23.7	1	1.3	0.0-6.9	5.0	0.1-24.9
**58**	13	5.8	3.1-9.8	21.0	11.7-33.2	6	4.3	1.6-9.0	15.0	5.7-29.8	7	8.9	3.6-17.4	35.0	15.4-59.2
**59**	7	3.1	1.3-6.4	11.3	4.7-21.9	3	2.1	0.4-6.1	7.5	1.6-20.4	4	5.1	1.4-12.5	20.0	5.7-43.7
**73**	5	2.2	0.7-5.1	8.1	2.7-17.8	4	2.8	0.8-7.1	10.0	2.8-23.7	1	1.3	0.0-6.9	5.0	0.1-24.9
**81**	5	2.2	0.7-5.1	8.1	2.7-17.8	3	2.1	0.4-6.1	7.5	1.6-20.4	1	1.3	0.0-6.9	5.0	0.1-24.9
**82**	1	0.4	0.0-2.5	1.6	0.0-8.7	0	0.0	0.0-2.6	0.0	0.0-8.8	1	1.3	0.0-6.9	5.0	0.1-24.9
**PHR/HR-HPVs**															
**66/68**	6	2.7	1.0-5.8	9.7	3.6-19.9	5	3.5	1.2-8.1	12.5	4.2-26.8	1	1.3	0.0-6.9	5.0	0.1-24.9
**PHR-HPVs**															
**53**	6	2.7	1.0-5.8	9.7	3.6-19.9	4	2.8	0.8-7.1	10.0	2.8-23.7	2	2.5	0.3-8.8	10.0	1.2-31.7
**LR/PHR-HPVs**															
**84/26**	2	0.9	0.1-3.2	3.2	0.4-11.2	1	0.7	0.0-3.9	2.5	0.1-13.2	1	1.3	0.0-6.9	5.0	0.1-24.9
**LR-HPVs**															
**6**	15	6.7	3.8-10.8	24.2	14.2-36.7	8	5.7	2.5-10.9	20.0	9.1-35.6	6	7.6	2.8-15.8	30.0	11.9-54.3
**11**	12	5.4	2.8-9.2	19.4	10.4-31.4	9	6.4	3.0-11.8	22.5	10.8-38.5	3	3.8	0.8-10.7	15.0	3.2-37.9
**42**	5	2.2	0.7-5.1	8.1	2.7-17.8	3	2.1	0.4-6.1	7.5	1.6-20.4	2	2.5	0.3-8.8	10.0	1.2-31.7
**70**	7	3.1	1.3-6.4	11.3	4.7-21.9	5	3.5	1.2-8.1	12.5	4.2-26.8	2	2.5	0.3-8.8	10.0	1.2-31.7
**72**	6	2.7	1.0-5.8	9.7	3.6-19.9	4	2.8	0.8-7.1	10.0	2.8-23.7	2	2.5	0.3-8.8	10.0	1.2-31.7
**40/61**	8	3.6	1.6-6.9	12.9	5.7-23.9	7	5.0	2.0-10.0	17.5	7.3-32.8	1	1.3	0.0-6.9	5.0	0.1-24.9
**43/44**	17	7.6	4.5-11.9	27.4	16.9-40.2	11	7.8	4.0-13.5	27.5	14.6-43.9	6	7.6	2.8-15.8	30.0	11.9-54.3
**54/55**	5	2.2	0.7-5.1	8.1	2.7-17.8	4	2.8	0.8-7.1	10.0	2.8-23.7	1	1.3	0.0-6.9	5.0	0.1-24.9
**57/71**	1	0.4	0.0-2.5	1.6	0.0-8.7	1	0.7	0.0-3.9	2.5	0.1-13.2	0	0.0	0.0-4.6	0.0	0.0-16.8
**X**	2	0.9	0.1-3.2	3.2	0.4-11.2	2	1.4	0.2-5.0	5.0	0.6-16.9	0	0.0	0.0-4.6	0.0	0.0-16.8

N: total number of times, which each genotype, was detected.

* Percentages referred to the total number of virus detected (223 viruses in the Total of lesions, 141 in the LASIL and 79 in HASIL).

** Percentages referred to the number of lesions infected by one or several genotypes (62 Total lesions, 40 LASIL and 20 HASIL).

CI95%: 95% confidence intervals used for estimate percentages.

In the majority of lesions, at least one HR-HPV was detected. Among all the lesions, HPV-16 was present in 37.1% (95% CI: 25.2%-50.3%), HPV-52 in 30.6% (95% CI: 19.6%-43.7%), HPV-51 in 22.6% (95% CI: 12.9%-35%), HPV-31 in 21% (95% CI: 11.7%-33.2%), and HPV-58 in 21.0% (95% CI: 11.7%-33.2%). Two cases (0.9%) were classified as an uncharacterized HPV type (HPV-X, 95% CI: 0.1%-3.2%), but we suspect that this classification was most likely related to the missed detection of known HPV types; i.e., it was probably not associated with an undiscovered HPV type (Table 2).

### Relationship between diagnoses and HPV genotypes

Among the LASIL samples, HPV-16 was not the most common genotype. The most frequent type was HPV-52 (9.2%; 95% CI: 5%-15.3%); this was followed by, in order of decreasing frequency, HPV-16 (8.5%; 95% CI: 4.5%-14.4%), HPV-43/44 (7.8%; 95% CI: 4%-13.5%), HPV-51 (7.1%; 95% CI: 3.5%-12.7%) and HPV-11 (6.4%; 95% CI: 3%-11.8%). HPV-18 was not detected in this group. Among the LASILs, HR-HPVs were detected more frequently than LR-HPVs (approximately 58.2% vs. 36.9%) and LR-HPV types were rarely identified as single infections (approximately 12.5%).

However, among the HASIL samples, HPV-16 was the most common genotype (12.7%; 95% CI: 6.2%-22%), followed by HPV-58 (8.9%; 95% CI: 3.6%-17.4%) and the group of HPV-31, HPV-52, HPV-6 and HPV-43/44 (7.6%; 95% CI: 2.8%-15.8%). HPV-18 was only detected in 2.5% (95% CI: 0.3%-8.8%). Also among the HASILs, HR-HPVs were detected more frequently than LR-HPVs (approximately 67.1% vs. 29.1%) a greater proportion of HR-HPVs were found in HASIL than in LASIL samples.

The only case of IC was found in a 67 year old male with a long history of Candidiasis and Cystoporidium infections. The sample from this individual also exhibited a double infection with HPV-16 and HPV-81.

The distribution of HPV genotypes and the analysis between pathology groups vs. HPV risk types is shown in Table 2.

**Figure 1:**
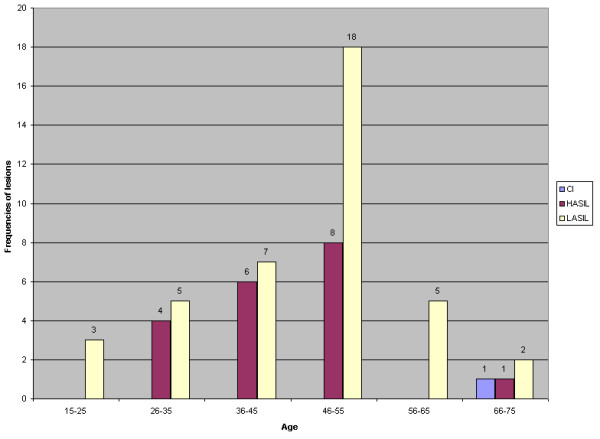
Chart showing the distribution of all cases according to their age and the frequency of each lesion.

Figure 1 shows a chart of the distribution of all cases according to patient age and the frequency of each lesion.

### Genotypes in co-infections

The percentages of multiple infections were very high: 80% in LASIL cases, 95% in HASIL cases, and 100% in IC cases. Multiple infections were not detected in benign lesions.

As shown in Table 3, among all the lesions with multiple HPV infections, the most common included four different genotypes (25.0%); similar frequencies were found for double genotype infections (21.2%) and triple genotype infections (19.2%). Five different genotypes were found in 17.3% of cases, and six genotypes were found in 13.5% of cases. More than six different genotypes were found in only two cases (3.8%). These results indicated a high prevalence of HPV co-infections.

**Table 3:** Pathological diagnoses and co-infection occurrence

	**N (%)**						
**Pathological diagnosis**	**2 HPV types**	**3 HPV types**	**4 HPV types**	**5 HPV types**	**6 HPV types**	**<6 HPV types**	**Total**
**LASIL**	8 (15.4)	7 (13.5)	5 (9.6)	5 (9.6)	5 (9.6)	2 (3.8)	32 (61.5)
**HASIL**	2 (3.8)	3 (5.8)	8 (15.4)	4 (7.7)	2 (3.8)	0 (0.0)	19 (36.5)
**IC**	1 (1.9)	0 (0.0)	0 (0.0)	0 (0.0)	0 (0.0)	0 (0.0)	1 (1.9)
**Total**	11 (21.2)	10 (19.2)	13 (25.0)	9 (17.3)	7 (13.5)	2 (3.8)	52 (100.0)

N: total of times, which each number, of genotypes was detected.

Percentage referred to total of co-infections (52).

Among the LASIL cases with multiple HPV infections, HPV-16 was the most common type found (9.0%; CI: 4.7%-15-2) followed by HPV-43/44, HPV-51 and HPV-52 (7.5%; CI: 3.7%-13.4%). HPV-11 occurred in 6.8% (CI: 3.1%-12.5%). Among the HASIL cases with multiple infections, the HPV genotype distribution was similar to that found in all the entire group of HASIL cases.

HPV co-infection distributions are shown in Table 4.

**Table 4:** HPV genotype distribution in coinfection cases

**Genotype found**	**LASIL**					**HASIL**				
	**N**	% *	**CI95%**	% **	**CI95%**	**N**	% *	**CI95%**	% **	**CI95%**
**HR-HPVs**										
**16**	12	9.0	4.7-15.2	37.5	21.1-56.3	10	12.8	6.3-22.3	52.6	28.9-75.6
**18**	0	0.0	0.0-2.7	0.0	0.0-10.9	2	2.6	0.3-9.0	10.5	1.3-33.1
**31**	6	4.5	1.7-9.6	18.8	7.2-36.4	6	7.7	2.9-16.0	31.6	12.6-56.6
**33**	2	1.5	0.2-5.3	6.3	0.8-20.8	4	5.1	1.4-12.6	21.1	6.1-45.6
**35**	3	2.3	0.5-6.5	9.4	2.0-25.0	2	2.6	0.3-9.0	10.5	1.3-33.1
**39**	3	2.3	0.5-6.5	9.4	2.0-25.0	1	1.3	0.0-6.9	5.3	0.1-26.0
**45**	6	4.5	1.7-9.6	18.8	7.2-36.4	2	2.6	0.3-9.0	10.5	1.3-33.1
**51**	10	7.5	3.7-13.4	31.3	16.1-50.0	4	5.1	1.4-12.6	21.1	6.1-45.6
**52**	10	7.5	3.7-13.4	31.3	16.1-50.0	6	7.7	2.9-16.0	31.6	12.6-56.6
**56**	4	3.0	0.8-7.5	12.5	3.5-29.0	1	1.3	0.0-6.9	5.3	0.1-26.0
**58**	6	4.5	1.7-9.6	18.8	7.2-36.4	7	9.0	3.7-17.6	36.8	16.3-61.6
**59**	3	2.3	0.5-6.5	9.4	2.0-25.0	4	5.1	1.4-12.6	21.1	6.1-45.6
**73**	4	3.0	0.8-7.5	12.5	3.5-29.0	1	1.3	0.0-6.9	5.3	0.1-26.0
**81**	3	2.3	0.5-6.5	9.4	2.0-25.0	1	1.3	0.0-6.9	5.3	0.1-26.0
**82**	0	0.0	0.0-2.7	0.0	0.0-10.9	1	1.3	0.0-6.9	5.3	0.1-26.0
**PHR/HR-HPVs**										
**66/68**	5	3.8	1.2-8.6	15.6	5.3-32.8	1	1.3	0.0-6.9	5.3	0.1-26.0
**PHR-HPVs**										
**53**	4	3.0	0.8-7.5	12.5	3.5-29.0	2	2.6	0.3-9.0	10.5	1.3-33.1
**LR/PHR-HPVs**										
**84/26**	1	0.8	0.0-4.1	3.1	0.1-16.2	1	1.3	0.0-6.9	5.3	0.1-26.0
**LR-HPVs**										
**6**	8	6.0	2.6-11.5	25.0	11.5-43.4	6	7.7	2.9-16.0	31.6	12.6-56.6
**11**	9	6.8	3.1-12.5	28.1	13.7-46.7	3	3.8	0.8-10.8	15.8	3.4-39.6
**42**	3	2.3	0.5-6.5	9.4	2.0-25.0	2	2.6	0.3-9.0	10.5	1.3-33.1
**70**	5	3.8	1.2-8.6	15.6	5.3-32.8	2	2.6	0.3-9.0	10.5	1.3-33.1
**72**	4	3.0	0.8-7.5	12.5	3.5-29.0	1	1.3	0.0-6.9	5.3	0.1-26.0
**40/61**	7	5.3	2.1-10.5	21.9	9.3-40.0	1	1.3	0.0-6.9	5.3	0.1-26.0
**43/44**	10	7.5	3.7-13.4	31.3	16.1-50.0	6	7.7	2.9-16.0	31.6	12.6-56.6
**54/55**	4	3.0	0.8-7.5	12.5	3.5-29.0	1	1.3	0.0-6.9	5.3	0.1-26.0
**57/71**	1	0.8	0.0-4.1	3.1	0.1-16.2	0	0.0	0.0-4.6	0.0	0.0-17.6
**X**	0	0	0.0-2.7	0.0	0.0-10.9	0	0.0	0.0-4.6	0.0	0.0-17.6

N: total number of times which each genotype was detected.

* Percentages referred to the total number of virus detected in each kind of lesion (133 virus in LASIL and 78 in HASIL lesions).

** Percentages referred to the number of lesions infected by one or several genotypes (32 LASIL and 19 HASIL lesions). CI95%: 95% confidence intervals used for estimate percentages.

## Discussion

This study included individuals with visible, HPV-related lesions in the anogenital area, assessed by careful inspection of the external genitalia and perianal area. Also, an intra-anal examination was performed with a digital rectal exam and high-resolution, flexible anoscopy, prior to an acetic acid stain of the mucosa. The population included was nearly universally HIV-infected; they were identified in an anal screening program conducted to prevent anal cancer.

In all the cases included in this study, a HPV genotyping assay was routinely performed on cytologic specimens. Some authors hold that the analysis of anal cytology for HPV may lead to an overestimation of genotype-specific associations with dysplasia [22]; however, given the multifocal characteristics of these lesions, we chose this procedure, because of it provided the ability to sample the entire anal canal and surface.

To our knowledge, this is the first prospective data publically available that estimated the type-specific prevalence of HPV in preinvasive anal lesions among patients that were HIV-seropositive in Spain. Among the results from this study, the main finding was the unexpectedly high presence of high-risk HPV genotypes in patients that were HIV-seropositive with anus pathology. The most frequent genotype found in all lesions was HPV-16 followed (in descending order) by HPV-52, HPV-43/44, HPV-6, HPV-51, HPV-31 and HPV-58. Four of these seven viruses belong to the species, group 9, of the genus, Alpha-papillomavirus, and five are known to be high-risk, oncogenic viruses [23,24]. This finding is important because it suggested that the anal lesions in this population might have had a greater predisposition to progression than initially expected. Furthermore, among those genotypes, we highlighted HPV-16, HPV-58, and HPV-31 because they showed an increased frequency in HASILs compared to LASILs.

The above suspicion was also noted by Machalek et al. in a recent meta-analysis. They calculated a theoretical rate of progression from HASIL to anal cancer of about one in 600 per year in men that had sex with another man (MSM) and were HIV-positive, in contrast, the rate was only one in 4000 per year in MSM that were HIV-negative [10]. Similarly, this risk for anal cancer has been described as twice as high among MSM with HIV-infections that among those without HIV-infections [3].

In this context, it is currently well known that individuals that are HIV-seropositive have an increased rate of anal epithelial pathology related to HPV infections compared to individuals that are HIV-seronegative [25-27]. In the series presented here, men outnumbered women by 15:1, with an average age of 46 years. All individuals were HIV-infected, and they were under treatment with highly active antiretroviral therapy (HAART). Among this cohort, it was relatively common to find concomitant infections, like syphilis and hepatitis B and/or C. This profile of patients was not surprising because for years, works by Daling 1982, Peters 1984, Croxson 1984, and Frazer 1986 have described the striking frequency of syphilis, anal warts, and ASIL in homosexual men, with or without AIDS [28-31].

Of note, it may be important that in our series of patients mostly belonged to a population born between 1958 and 1992. This age group may be related to the probability of survival with AIDS and the possible loss of HIV-infected patients in previous years; thus, relatively younger men were more highly represented compared to those born between 1938 and 1957. This fact can be explained by the undoubted benefit of HAART, which was introduced in 1996.

The results of this paper were consistent with those reported recently, which found the most frequent genotype was the HPV-16, and that HIV-seropositive patients exhibited the highest proportion of multiple HPV co-infections [7,15,19,22,32]. However, we note that there were some differences between the HPV genotypes found in our study and those found in other populations. These differences may be explained by geographical diversity. For example, in the French EdiTH V study the most frequent HPV genotypes found among HIV-seropositive cases were HPV-16 (59.6%), HPV-18 (15.4%), HPV-68 (13.4%), and HPV-51 and HPV-52 (11.5%) [20]. Similarly, a few years earlier, Orlando et al. reported in Italy that the genotypes identified most frequently were HPV-16 (21.69%), HPV-6 (18.16%), HPV-11 (16.27%), HPV-58 (5.19%), HPV-30 (4.72%), HPV-26 (4.24%) and HPV-18 (3.07%) [19].

The frequency of cases with the HPV-18 genotype in our study was not as high as that published in other international series [32,33]. We had found a similar discrepancy in previous work on intraepithelial neoplasm and invasive carcinomas of the cervix. However, this lower frequency of HPV-18 was consistent with other studies conducted in Spain where the HPV-18 type did not appear to be as common in the general population as it was in other countries [34,35].

As stated above, the patients with HIV-infections were more likely to have multiple infections and more frequently had oncogenic HPV types than those without HIV-infections. In our series, 83.9% of lesions had multiple infections, which was a higher rate than those reported by Palesfsky and Hesson [26,27]. Hesson et al. also found that multiple HPV infections occurred more frequently among patients with low levels of lymphocytes CD4. Other authors that found similar results linked them to the immunodeficiency [20].

The most frequent co-infection pattern was multiple infections with four different HPV types (25%). Although we sought to find a dominant pattern of HPV types in concurrent infection, we only observed random patterns.

Finally, to date, there is no established consensus regarding the best treatment for ASILs. The treatments reported for HASILs, both medical and surgical, have not been standardized. Studies that investigated the preventive and therapeutic effects of existing HPV vaccines have reported that vaccines can reduce persistent anal HPV infections and HASILs among immunized MSM. However, the use of vaccines in preventing these lesions in immunocompromised individuals, as expected, has been ineffective or yielded ambiguous results [36]. Therefore, it will be necessary to find new therapeutic strategies. Some new strategies are under currently development, such as the use of RNA aptamers selected against the HPV-16 oncoprotein E7. In the future, new strategies may play an important role in the management of these patients [37].

## Conclusion

In summary, this study showed that HPV-16 was the prevalent HPV subtype in a Spanish population. HPV-16 was associated with all grades of anal dysplasia and anal squamous carcinomas. Also, the prevalence of high-risk carcinogenic HPV genotypes in patients with anal pathology and at risk for HIV infection was remarkable. Therefore, further studies that include a larger number of samples, particularly samples with IC are needed to evaluate the potential influence of these HPV genotypes in the appearance of anal carcinomas.

### Study limitations

The present study had some limitations. First, the study had a retrospective design, which relied on medical records. This study design limited the available data to the information contained in the medical charts. The individual’s sexual habits and race/ethnicity were not noted in the chart. This cohort included a population of mostly Caucasian, urban, HIV-seropositive men that were treated in a public hospital; therefore, our findings may not be generalize to other populations.

Second, the samples were collected from only one hospital; thus, it may be difficult to generalize the results to the entire population of Spain. However, this hospital was a reference centre that served patients from many districts and municipalities of the Region of Madrid.

Third, although more than 40 anogenital HPV types exist, only 33 were investigated in this study. However, these 33 included all the HPV genotypes that are typically implicated in the initiation of anal cancer.

Fourth, the modest sample size limited the statistical power for our analyses. However, it is difficult to acquire this kind of samples in Spain; therefore, these results provide valuable information. In the future, it would be of interest to obtain more samples for further studies.

## Abbreviations

ASIL: Anal squamous intraepithelial lesion; HPV: Human papillomavirus; HR-HPV: HPV type associated with a high risk of carcinogenesis; PHR-HPV: HPV type associated with a high probability of risk of carcinogenesis; LR-HPV: HPV type associated with a low risk of carcinogenesis; BL: Benign lesion; LASIL: Low grade anal squamous intraepithelial lesion; HASIL: High grade anal squamous intraepithelial lesion; IC: Invasive carcinoma.

## Competing interests

The authors declare that they have non-financial competing interests.

## Authors’ contributions

BGE participated in the design of the study, global data acquisition, analysis and preparation of the first draft. EMR participated in writing the first draft, critically revised the first and final drafts, participated in the data acquisition and participated in planning the study design. EAF conceived the design of the study and participated in the surgical pathological data acquisition and analysis. All authors read and approved the final manuscript.
